# Adoption of a Postoperative Pain Self-Report Tool: Qualitative Study

**DOI:** 10.2196/33706

**Published:** 2022-04-26

**Authors:** Bram Thiel, Inez Iao, Joris Smid, Emmy de Wit, Seppe Koopman, Bart Geerts, Marc Godfried, Cor Kalkman

**Affiliations:** 1 Department of Anesthesiology OLVG Hospital Amsterdam Netherlands; 2 Department of Services and Solutions Delivery Philips Benelux Amsterdam Netherlands; 3 Department of Cardiology OLVG Hospital Amsterdam Netherlands; 4 Faculty of Science Athena Institute Vu University Amsterdam Netherlands; 5 Department of Anesthesiology Maasstad Hospital Rotterdam Netherlands; 6 Department of Intensive Care Spaarne Gasthuis Hospital Haarlem Netherlands; 7 Department of Anesthesia and Intensive Care University Medical Centre Utrecht Utrecht Netherlands

**Keywords:** innovation, eHealth adoption model, mobile health, pain, self-report, perioperative medicine, postoperative pain, surgery

## Abstract

**Background:**

With electronic technologies, patients are provided with tools to easily acquire information and to manage and record their own health status. eHealth interventions are already broadly applied to perioperative care. In a similar way, we aimed to utilize a smartphone application to enable postoperative patients to partially self-manage their postoperative pain. The results of a previously performed proof-of-concept study regarding the application were promising, and nurses as well as patients were optimistic regarding this innovative mobile application. Nevertheless, in reality, it appears that the usage and overall implementation of this application have stagnated since its introduction. Problems with innovation adoption are not novel; various studies have been conducted to explore the reasons for low implementation success of eHealth applications and indicated that adoption is influenced by multiple organizational factors. This study investigated the influence of these organizational factors on the adoption process, aiming to provide more insight in the dos and don’ts for implementing eHealth in the working processes of hospital care.

**Objective:**

This study aimed to provide insight in how to successfully implement a technological eHealth innovation in a general nonacademic hospital.

**Methods:**

A qualitative study was conducted to explore organizational factors affecting the innovation adoption process. Data were collected by conducting semistructured one-on-one interviews with 11 stakeholders. The data were analyzed using thematic analysis identifying overarching themes.

**Results:**

Absorptive capacity, referred to as an organization’s dynamic capability pertaining to knowledge creation and utilization that enhances an organization’s ability to gain and sustain a competitive advantage, was regarded as the most influential factor on the application’s adoption. Accordingly, it appeared that innovation adoption is mainly determined by the capability and willingness to assimilate and transform new information into productive use and the ability to absorb a novel innovation. Absorptive capacity was found to be influenced by the innovation’s benefit and the sense of ownership and responsibility. Organizational readiness and management support were also regarded as essential since absorptive capacity seemed to be mediated by these factors. The size of the hospital influenced eHealth adoption by the amount of resources available and by its organizational structure.

**Conclusions:**

In conclusion, absorptive capacity is essential for eHealth adoption, and it is mediated by management support and organizational readiness. It is recommended to increase the degree of willingness and ability to adopt an eHealth innovation by enhancing the relevance, engaging stakeholders, and assigning appropriate leaders to offer guidance.

## Introduction

To date, hospital care, admission potential, urgent and nonurgent care, and health care professionals are under pressure due to problems such as the aging population, the rising prevalence of chronic comorbid diseases, and overall increasing health care costs [1,2]. On top of these already existing problems came the COVID-19 pandemic. These growing demands accelerate the surge in eHealth innovations [3-5].

With electronic technologies, patients are provided with tools to easily acquire information and to manage and record their own health status. eHealth interventions are already broadly applied to perioperative care (eg, remote monitoring, educational websites, and telerehabilitation) [6,7]. In a similar way, we aimed to utilize an eHealth tool (the OLVG Pain App) with the objective to improve the efficiency and quality of postoperative pain management. This eHealth tool enables the patient to partially self-manage their postoperative pain, as they record their own postoperative pain intensity in their electronic medical record. The results of a previously performed proof-of-concept study regarding the OLVG Pain App were promising, and nurses as well as patients were optimistic regarding this innovative mobile application [8].

Nevertheless, in reality, it appears that the usage and overall implementation of this application have stagnated since its introduction. Problems with innovation adoption are not novel; various studies have been conducted to explore the reasons for low implementation success of eHealth applications and indicated that adoption is influenced by multiple organizational factors such as technological knowledge and skills, financial aspects, social and organizational support, and a lack of education and training [9-13]. Therefore, the aim of this study was to provide insight on how to successfully implement a technological eHealth innovation in a general nonacademic hospital. Accordingly, the research question was: “How can the adoption process of the PainApp be understood with regards to the organizational factors within a general hospital?”

## Methods

### Study Design

A qualitative study was conducted between March 1, 2020, and July 31, 2020. Stakeholders involved with the development and implementation process of the application were interviewed to provide an in-depth understanding of how the context of a general hospital can facilitate or hamper the adoption of eHealth. The study was conducted and is reported according to the CONSORT-EHEALTH (Consolidated Standards of Reporting Trials of Electronic and Mobile HEalth Applications and onLine TeleHealth) checklist (V.1.6.1) [14], the consolidated criteria for reporting qualitative research (COREQ) [15], and the “Qualitative research: standards, challenges, methodological guidelines” by Malterud [16].

### Recruitment

For this study, the perceptions of stakeholders active within the departments of Information Technology (IT), Electronic Medical Record (EMR), Anesthesiology, and Nursing Staff Convention from 2 hospitals were investigated. Both OLVG Hospital and Maasstad Hospital are general hospitals providing surgical care for 23,000 patients annually.

The sample size was determined by the concept of “information power,” which depends on the relevance of the participants included [17,18]. Based on this premise and similar studies, 11 participants were considered satisfactory, as the selected participants were highly informative and significant actors in the innovation procedure (Table 1, participant characteristics). Participants from specific departments within the OLVG Hospital and Maasstad Hospital were provided by personal contacts from the supervisors of this study.

**Table 1:** Participant characteristics.

Identification number	Hospital	Position	Age (years)	Gender	Work experience (years)	Technical background
P1	OLVG Hospital	Anesthetist	52	M^a^	17	6 years of postoperative home monitoring and digitalized preoperative screening
P2	OLVG Hospital	Nurse, application specialist	42	M	18	6 years as an application specialist for the EHR^b^
P3	Maasstad Hospital	Anesthetist	39	M	6	1 year as a CMIO^c^
P4	OLVG Hospital	Neurologist	39	M	6	3 years as an innovation specialist, human-centered design, and eHealth implementation
P5 LKru	OLVG Hospital	Department manager	45	M	10	5 years as a manager of the EHR and patient participation
P6	OLVG Hospital	Nurse team leader	37	F^d^	15	3 years as a key user of the EPIC EHR
P7	OLVG Hospital	Department manager	-^e^	M	-	6 years as a manager of the ICT^f^ department
P8	OLVG Hospital	Nurse team leader	-	F	28	10 years as a nurse team leader in the neurosurgery department
P9	OLVG Hospital	Operational manager	-	F	39	4 years as a manager of the neurosurgery business unit
P10	OLVG Hospital	Pulmonologist	62	M	30	2 years as a CMIO
P11	Maasstad Hospital	Clinical computer scientist	34	M	5	Specialty connectivity between medical devices and the EHR

^a^M: male.

^b^EHR: electronic health record.

^c^CMIO: Chief Medical Information Officer.

^d^F: female.

^e^Participant did not respond to the extra questions for age and years of work experience.

^f^ICT: information communication and technology.

### Data Collection

The data were collected during semistructured one-on-one interviews performed by a research student (IK). The interviews were structured by the following topic questions: size of the hospital, top management support, organizational readiness, and centralization in decision-making and absorptive capacity. These topics were based on pre-established themes derived from the eHealth Adoption Model (eHAM) [19]. The eHAM combines elements of the diffusion of innovation theory and the technology-organization-environment framework, since these form a theoretical base of innovation in various sectors [20].

The interviews were conducted in Dutch; the interview guide was originally formulated in Dutch, followed by a translation into English for the purpose of this report (Multimedia Appendix 1). Prior to the interview, participants received some general information concerning the research topic, and informed consent was requested from the interviewee to allow for audio recording of the interview. The interview commenced with introductory questions, with which the general opinion on eHealth innovations of eeeof the participant was established. After this introductory phase, topic questions regarding the concepts of the eHAM model were asked to explore their experiences with and perspectives on the influences of these factors. Lastly, specific closing questions recapping the themes were asked to evaluate the importance of each organizational factor. Furthermore, interviews were conducted online through Skype, Zoom, or FaceTime due to COVID-19 measures.

### Data Analysis

Data collection and analysis progression were discussed during regular meetings with the researchers BT and JS and the research student IK. A thematic framework approach was utilized in order to analyze the qualitative data [21,22]. First, the audio recordings of the interviews were transcribed verbatim in Dutch. As for the validity, the interpretations of the interview were sent to the respective participants to check whether the interviews were well understood and in line with their perspectives. After familiarization with the data, the transcripts were coded with an English coding guide that was developed according to the eHAM model. The coding guide included the 5 main concepts regarding the organizational factors, which were further differentiated into subconcepts. After coding, overarching themes and patterns were identified and labelled within each concept.

### Ethics Approval

This study was conducted as part of the “Closing the loop” project approved by the Advisory board for Science and Research (ACWO) OLVG Hospital on December 30, 2019, with registration number WO 19.167.

## Results

### Themes

The results were derived from stakeholder interviews and yielded a total of more than 40 themes that are related to the eHAM [19]. For a visualized overview of all identified and related themes, see Multimedia Appendix 2. Overall, the stakeholders considered absorptive capacity, top management support, and organizational readiness as the most essential factors for the adoption of eHealth innovation. For interview data, see Multimedia Appendix 3. Moreover, the remaining 2 factors (ie, size of the hospital and centralization in decision-making) were considered as generic influences and are therefore briefly discussed.

### Absorptive Capacity

Absorptive capacity appeared to be of great importance. Absorptive capacity refers to an organization’s “dynamic capability pertaining to knowledge creation and utilization that enhances an organization’s ability to gain and sustain a competitive advantage” [23].

The majority of stakeholders stated that the individual’s willingness is an essential aspect that can affect the degree of absorptive capacity. Next to the willingness to absorb new information, the ability to do so is also essential for successful innovation adoption.

Altogether, the participants mentioned that spreading new knowledge is more effective when done repetitively face-to-face, than, for instance, digitally through newsletters or by email. Clinical lessons and pilot tests can also be used to effectively introduce innovations or increase the skills of employees.

Another important aspect mentioned to influence absorptive capacity is the culture within the hospital, as a culture that is more “open” and stimulating in accepting new information can enhance the adaptability and flexibility of the organization.

Furthermore, this willingness and ability to adopt certain innovations also appeared to depend on various themes, such as personal characteristics, the context, and whether a sense of ownership and responsibility is present with the individual. The influence of personal characteristics on absorptive capacity became evident as 9 of 11 participants noted that features such as age (or generation), affinity with technology, and being an early or late adopter can affect whether an individual absorbs an innovation. Accordingly, a team leader stated:

When talking about absorptive capacity, it also depends a lot on the type of person. How much information can you assimilate? Are you theoretical or more practical? How old are you? Do you have an affinity for innovation and technology?

P6, team leader

Additionally, 6 (P9, P1, P4, P5, P3, P6) participants felt that hospital employees are commonly not very capable of acquiring, assimilating, transforming, and connecting new information to existing knowledge for productive use. They believed that hospital employees are extremely programmed to conform to protocols, which limits the potential for adoption and particularly affects their ability to embed innovations into practiced daily routines. An essential remark made by all participants is that the context in which the innovation adoption takes place has a significant role in whether individuals are willing to absorb it. It became clear that the severity or urgency of the problem that the innovation addresses and responds to should be sufficiently experienced by those who need to use the innovation.

In addition, all participants mentioned that the innovation’s relevance is a key factor for successful innovation adoption and that this mainly depends on whether there is a substantial benefit to fulfill the need of the user. Another theme that emerged from 6 interviews was that the innovation should induce a sense of ownership in order for employees to truly adopt the eHealth innovation.

### Top Management Support

Next to the absorptive capacity, the support of top management also appears to be of great influence, as managers are recognized to be responsible for delivering the required resources to facilitate innovation adoption. An anesthetist described the top management’s influence accordingly:

I think they certainly have an impact. They have to provide the money to be able to introduce eHealth tools such as the OLVG Pain app and support ICT and so on. Thus, if they say we won’t be doing it, then it won’t happen.

P3, anesthetist

In addition, top management also seems to be responsible for arranging employees and project structures for realization. All participants agree that commitment from the top and a supportive vision of innovations should be present for successful eHealth adoption. More importantly, one of the team leaders stated that all management layers should be committed, in particular the team leaders, since they are practically active at the site of implementation:

You want it to be implemented in the workplace, so you should focus on there. The employees have no idea of what is happening in the top of the organization. If it is transmitted by a team leader, then they will believe and follow it.

P8, team leader

### Organizational Readiness

Most of the participants experienced that the actual IT infrastructure was adequately present to facilitate technological developments. However, P6 did mention that the hospitals lag behind some current technologies due to limited financial resources and complex privacy concerns:

For example, we still do not have a blood pressure meter that automatically sends the data into the patient data management system (PDMS) via Wi-Fi. Nowadays, it must be possible to automatically Bluetooth or Wi-Fi everything to the system; why is it not possible for us to realize this?

P6, team leader

Some participants supported this decision as they believed that hospitals are not supposed to compete with third-party app developers:

I don't think we'll be any better at it, because the hospital is good at treating patients and our core business is not building an app.

P5, department manager

### Financial Readiness

The majority of participants mentioned that they are aware that hospitals have relatively little financial resources. Furthermore, most of the participants recognized that the financial readiness for eHealth innovations mainly depends on the innovation’s significance and potential for success. P6 described this accordingly:

Yes, if an innovation doctor says: “Yes we have to do this and I know it costs 100 thousand euros,” the innovation committee can still object by saying that there is no money for that, because it has to be spent on new patient beds, as that is simply more important than that innovation.

P6, team leader

### Size of the Hospital

Most of the participants agreed that the size of the hospital’s influence on eHealth implementation is associated with the available resources and the organizational structure. For instance, an IT employee mentioned that a larger hospital allows for more available resources, which in turn increases the organizational readiness for the adoption of eHealth innovations. On the other hand, a larger hospital appears to have more management layers and is therefore considered to be more bureaucratic in its structure. One of the participants (P5) stated correspondingly:

So yes, you have resources, but then again you are so big and bureaucratic that the speed of implementing innovations really slows down.

P5, department manager

### Centralization in Decision-making

All participants agreed that centralization is required for a structured overview to focus on hospital-wide interests and a fairer selection procedure of all projects. P5 described that a centralized group can positively influence eHealth adoption by providing guidance and resources:

That central group helps with getting a sharp picture of whether the innovation really matters and if that is what we want, and then they’ll help bridge the connection with those who can support, build, implement, and train it.

P5, department manager

## Discussion

### Principal Findings

**Figure 1:**
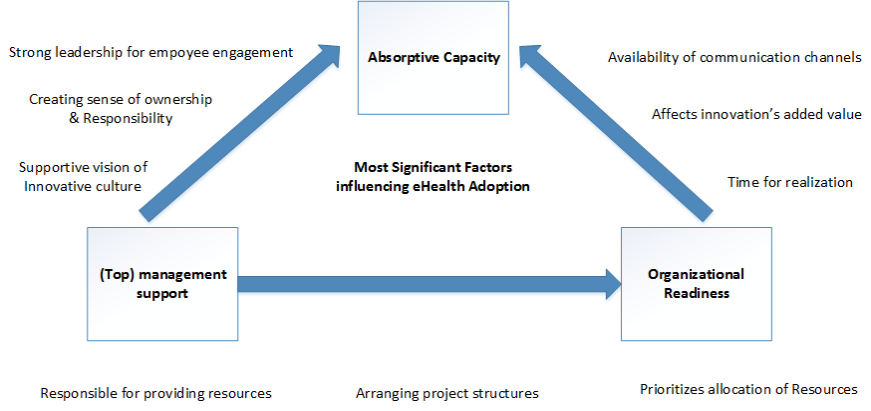
Absorptive capacity in relation to mediating factors.

The results of this study demonstrate that absorptive capacity is regarded as the most important factor influencing eHealth adoption in a general hospital. In addition, the degree of absorptive capacity is predominantly mediated by the amount of management support and the organizational readiness (Figure 1). However, the size of the hospital and centralization in decision-making are rather generic influences on the innovation process. Previous studies show supportive findings regarding the importance of absorptive capacity in influencing eHealth adoption [24-27]. We found that “being attached to follow fixed guidelines” (ie, working by following strict protocols), “context” (ie, the way urgency and relevance of a specific issue push an innovation), and the absence of a sense of ownership and responsibility affected absorptive capacity. Being a “family owner” could contribute to the willingness for innovation and therefore could positively influence absorptive capacity [28]. Next to highlighting the importance of absorptive capacity itself, we also reveal that both management support and organizational readiness mediate this factor. Numerous other studies also present that absorptive capacity is influenced by organizational structure, culture, and communication [27,29-31].

Our findings regarding organizational readiness are in accordance with other studies suggesting that organizations with a favorable environment, the structure, and the required resources are more prone to absorb innovations [32,33].

For financial readiness, we found that a hospital’s limited budget could be a barrier but would not be a determinant of the innovation’s actual success. It seemed that, rather, the innovation’s importance and potential to truly improve the quality of care would eventually determine the availability of financial resources.

The size of the hospital was found to influence the innovation process, as a larger size is positively associated with a greater amount of organizational resources, which in turn can facilitate better innovation implementation. This is in line with previous literature indicating that a greater hospital size influences the likelihood of successful adoption [34]. In contrast to this, our findings suggest that a greater size could also lead to a more bureaucratic structure and therefore even hamper innovation adoption despite its possession of more resources.

As for the centralization of decision-making, it became clear that this organizational factor differed in influence depending on the specific stage of the innovation process. Despite the potential positive influence of an acknowledgeable centralized group in guiding the innovation implementation, the stakeholders in our study ranked this factor as one of the least influential for successful adoption.

### Limitations

This study has several limitations; despite the recruitment of stakeholders from 2 different hospitals, the results and key messages of this study are mainly valid for the OLVG specifically, as the great majority of interviewees came from this hospital. This might have limited the acquirement of in-depth details on certain factors. The use of a conceptual model enabled this study to provide more insights into how the organizational factors influence adoption and to structure an overview of how these different factors relate to each other in terms of prioritization. However, the inclusion of such an extensive number of organizational factors may also have hampered a deeper understanding and exploration of each factor’s influence on its own. Accordingly, this is also the reason why the hospital’s “communication structures” have only been addressed briefly in our study and hence, lacks thoroughness in the findings.

### Recommendations for Practice

The results revealed that adoption was prominently lacking on the innovation’s benefit and on the sense of ownership and responsibility, which in turn negatively affected the absorptive capacity. Therefore, we suggest 3 focal points for policy.

The first focal point follows the “Quadruple Aim criteria” as a concept that focuses on examining the innovation according to the following 4 aims: lower cost of care, improved patient care, better health outcomes, and improved staff experience [35].

The second focal point increases the absorptive capacity from various aspects by using the value-sensitive design approach to engage all stakeholders from the beginning, creating a sense of ownership and responsibility [36].

The third focal point relates to our finding that management has a major influence on organizational culture and thus, also on the absorptive capacity. Accordingly, appropriate leaders who are truly able to stimulate an innovation-friendly learning culture should engage stakeholders. Therefore, the allocation of ambassadors or managers can support in overcoming resistance to change concerning stakeholder engagement and creating the innovation’s benefit.

### Conclusions

This study provides insight in how to successfully implement an eHealth innovation in a general hospital. The most important factor influencing eHealth adoption was absorptive capacity, which was mainly determined by the innovation’s urgency and relevance, and a sense of ownership and responsibility. Additionally, we revealed that absorptive capacity is mediated by management support and organizational readiness. Three focal points for successful eHealth adoption are enhancing the innovation’s relevance, adequately engaging stakeholders from the start, and allocating ambassadors or managers to support stakeholder engagement and to offer proper guidance and training.

## Appendix

Multimedia Appendix 1 Interview guide.

Multimedia Appendix 2 Overview of the identified themes.

Multimedia Appendix 3 Interview data.

## Abbreviations

ACWO: Advisory board for Science and Research
CONSORT-EHEALTH: Consolidated Standards of Reporting Trials of Electronic and Mobile HEalth Applications and onLine TeleHealth
COREQ: consolidated criteria for reporting qualitative research
eHAM: eHealth adoption model
EMR: electronic medical record
IT: information technology
PDMS: patient data management system
