# Supplementary material for: Adoption of a Postoperative Pain Self-Report Tool: Qualitative Study
Source: JMIR Hum Factors. 2022 Apr 26;9(2):e33706. doi: 10.2196/33706 (PMC9092239; doi:10.2196/33706)
Supplement: Multimedia Appendix 1 [file humanfactors_v9i2e33706_app1.docx]

Supplemental material I (Interview Guide)

**Interview/Topic guide - English**

1. Introduction of study/interview

1a. Study about the organizational factors that influence the PainApp (or another eHealth innovation within the hospital)

1b. Explaining the theory/conceptual model, the process of the interview, and the concepts that will be discussed: Size of hospital, Top management support, Organizational readiness, centralization in decision making and absorptive capacity

1. Introductory questions

How many years of working experience do you have in the hospital?

Can you tell something about your function and the work you do?

Are you involved in the development process of the PainApp (or another eHealth innovation)?

If yes, what is your role?

Do you have experience in digital applications as a medical employee?

How proficient do you consider yourself with these digital applications?

1. Concepts

3a. Size of hospital

Do you think that the size of the hospital influences the implementation/adoption of the PainApp?

3b. Top management support

To what extent do you think that top management influences the adoption of the PainApp?

Do you think that both positions contribute equally to the success of an eHealth innovation? To what extent does their influence differ?

3c. Organizational readiness

Technological readiness:

Are there sufficient technological resources (e.g. IT system/infrastructure) and skills (knowledge/IT specialists) present within the hospital?

Are these resources and skills utilized for the PainApp?

Financial readiness:

Are there sufficient financial resources (i.e. money) to realise the implementation/adoption of the PainApp?

Are there sufficient financial resources spent on/provided for eHealth innovations?

3d. Centralization in decision-making:

How do you experience the decision-making within the OLVG/hospital?

To what extent does this type of decision-making influence the adoption/implementation of the PainApp?

3e. Absorptive capacity

How do you experience the spread of novel knowledge/information in the OLVG/hospital?

Are there sufficient communication structures for new information? Are these adequately provided?

Do you think that the employees have sufficient knowledge/skills to use the PainApp? Why do you think that?

1. Closing

Which of the discussed themes/concepts (organizational factors) do you consider most important for the implementation/adoption of an eHealth innovation.
