# Supplementary figures and images for: Adoption of a Postoperative Pain Self-Report Tool: Qualitative Study
Source: JMIR Hum Factors. 2022 Apr 26;9(2):e33706. doi: 10.2196/33706 (PMC9092239; doi:10.2196/33706)

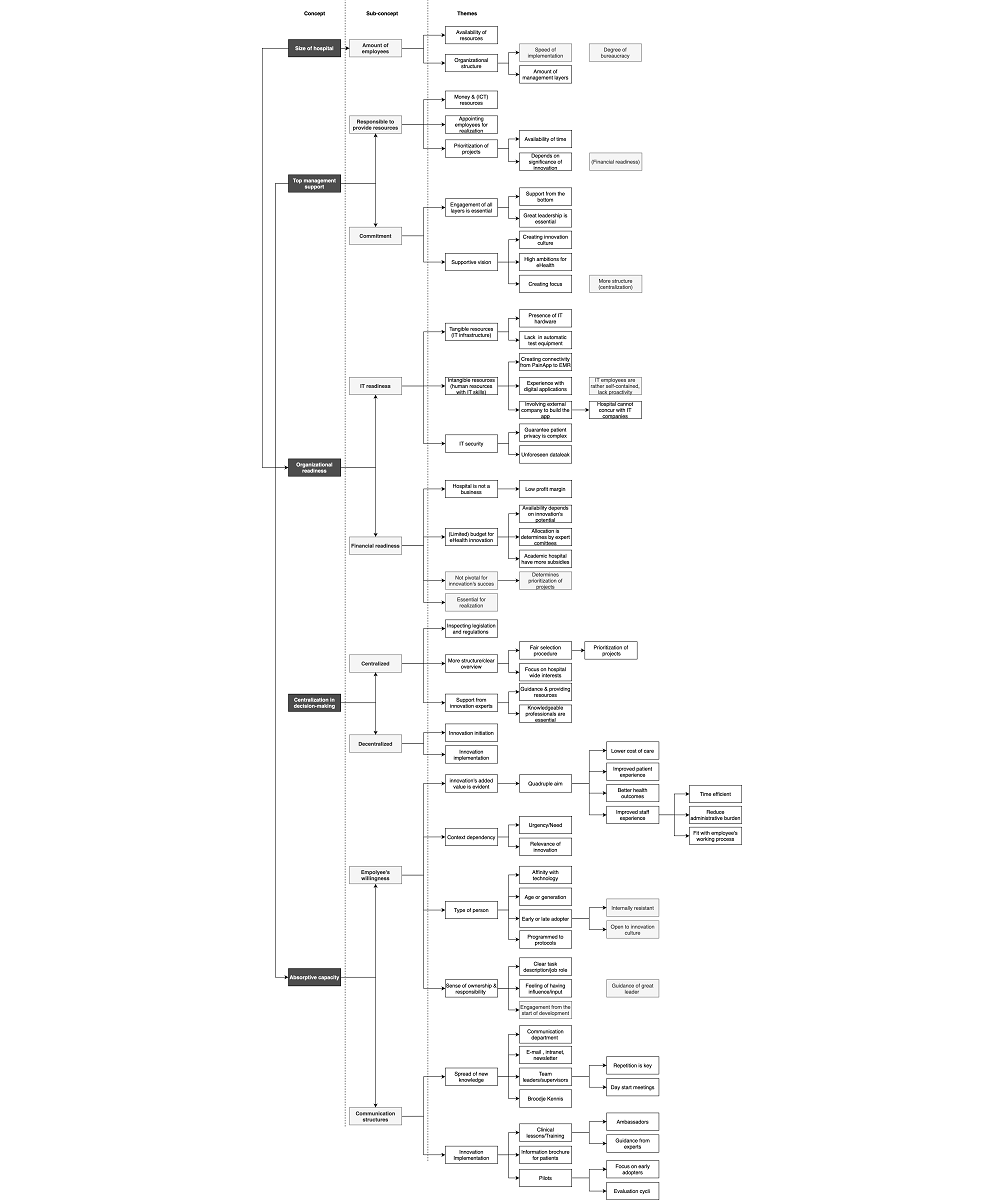

Supplement: Multimedia Appendix 2 [file humanfactors_v9i2e33706_app2.png]
